# Validating the architecture of cognitive distortions in Russian discourse using artificial intelligence and bootstrap analysis

**DOI:** 10.3389/fpsyg.2026.1740864

**Published:** 2026-02-23

**Authors:** Igor Gajniyarov

**Affiliations:** N.N. Krasovskii Institute of Mathematics and Mechanics of the Ural Branch of the Russian Academy of Sciences, Yekaterinburg, Russia

**Keywords:** cognitive distortions, cognitive behavioral therapy, large language models, digital mental health, computational mental health, network psychometrics, large-scale text analysis, discourse analysis

## Abstract

**Introduction:**

Cognitive distortions—systematic thinking biases linked to depression and anxiety—frequently co-occur in clinical practice, yet empirical evidence for their interaction patterns remains limited, particularly in non-Western populations where cognitive patterns may vary cross-culturally.

**Methods:**

We analyzed 249,414 Russian-language texts from social media and forums (2020–2024) using two large language models achieving substantial expert agreement (κ = 0.73). Association rule mining identified co-occurrence patterns; network stability was evaluated through bootstrap validation and split-half reliability analysis.

**Results:**

Analysis identified 443,447 distortion instances across 18 categories (M = 1.78 per text). All-or-nothing thinking showed highest prevalence (15.5%), followed by overgeneralization (14.2%) and catastrophizing (11.4%). Network analysis identified a stable core of 11 nodes (bootstrap stability ≥95%) and 2 peripheral, less stable nodes (Fairness 93%, Fortune Telling 60.8%). The resulting 13-node network was connected by 35 significant associations (density = 0.449, clustering = 0.598). Five distortions failed stability thresholds (< 60%) and were excluded. Strongest dyadic pattern: all-or-nothing/catastrophizing (*lift* = 1.96, *p* < 0.001). These two distortions appeared each in 67% of all significant triadic patterns. Personalization demonstrated highest degree centrality (degree = 10). Split-half reliability was high (r = 0.943).

**Discussion:**

Automated classification revealed hierarchically organized co-occurrence network in Russian-language discourse with personalization as primary hub and all-or-nothing/catastrophizing forming densely connected core. Findings suggest cluster-based interventions may be effective for Russian-speaking populations, though cross-cultural replication is required to distinguish universal mechanisms from cultural patterns. Cross-sectional design and single-language sample limit causal inference and generalizability.

## 1 Introduction

Cognitive distortions—systematic thinking errors contributing to psychological distress—frequently co-occur within the same discourse (appearing together in single texts) and may operate as interconnected systems. Clinical observations suggest that addressing one distortion may be accompanied by changes in others, though whether this reflects compensatory mechanisms, shared cognitive processes, or measurement artifacts remains an empirical question. However, empirical evidence systematically mapping these interaction patterns remains limited.

Large language models now enable population-scale pattern analysis of naturalistic cognitive expression. Early machine learning approaches demonstrated feasibility of automated distortion detection (Shickel et al., 2019), while recent advances show LLM accuracy in detecting psychological constructs (r = 0.59–0.77) outperforms dictionary-based methods (r = 0.20–0.30) (Rathje et al., 2024). However, systematic application to mapping cognitive distortion networks remains unexplored. Research reveals distinct cognitive distortion patterns: self-serving distortions associate with aggression while self-debasing patterns link to depression (Barriga et al., 2008; Leung and Poon, 2001). Recent computational studies demonstrate that individuals with depression express more distorted thinking patterns in social media discourse (Bathina et al., 2021), providing empirical foundation for automated detection methods.

Despite advances in detecting individual cognitive distortions (Kim et al., 2025; Settanni et al., 2025), their co-occurrence patterns in naturalistic contexts remain unmapped. Recent evidence linking distortion frequency with depression severity in clinical transcripts (Lalk et al., 2024) underscores the need for systematic pattern analysis. Methodological constraints include small sample sizes and reliance on self-report measures.

Cognitive distortions operate as transdiagnostic mechanisms across psychiatric conditions. Catastrophizing represents a core process in anxiety disorders, including panic disorder, generalized anxiety disorder, and obsessive-compulsive disorder (Gellatly and Beck, 2016), while emotion regulation difficulties—including catastrophic thinking patterns—maintain psychopathology across anxiety, depression, substance use, eating disorders, and borderline personality disorder (Sloan et al., 2017). Transdiagnostic frameworks emphasize shared processes over categorical boundaries (Norton and Paulus, 2017).

Network models in psychopathology identify “bridge symptoms” connecting disorder clusters (Buchwald et al., 2024). Similarly, certain cognitive distortions may function as bridge nodes. This study investigates whether specific distortions demonstrate such gateway properties through co-occurrence network position.

This exploratory study analyzes cognitive distortion co-occurrence patterns in Russian-language online discourse (*N* = 249,414 texts) to test architectural generalizability beyond Western (WEIRD) samples. Russian discourse provides a critical test case: cross-cultural attribution research shows collectivistic contexts favor external attributions (Oyserman et al., 2002), potentially reducing Personalization centrality compared to Western samples. Additionally, platform anonymity may elicit distinct disclosure patterns absent from clinical settings. Rather than testing specific predictions, an informed exploration approach is adopted—using established CBT theory to interpret emergent patterns while remaining open to novel configurations. This addresses the WEIRD generalizability gap in cognitive models.

### 1.1 Current study

Given the limited empirical data on cognitive distortion networks in non-Western populations, this study employs an exploratory design. Adopting an inductive approach, the investigation is guided by three research questions regarding the architecture of cognitive distortions:

- **RQ1 (Dyadic co-occurrence):** Do cognitive distortions co-occur non-randomly in naturalistic discourse, and which pairs demonstrate the strongest associations?
- **RQ2 (Triadic configurations):** Do stable three-way combinations emerge, forming complex cognitive configurations beyond simple pairs?
- **RQ3 (Network centrality):** Is centrality distributed heterogeneously, with certain distortions functioning as structural hubs within the network?

## 2 Methods

### 2.1 Goals and tasks of research

The primary goal was to identify co-occurrence patterns among cognitive distortions in Russian-language online discourse through automated classification and network analysis, addressing three research questions regarding dyadic patterns (RQ1), triadic configurations (RQ2), and network centrality (RQ3).

### 2.2 Research design

This naturalistic observational study analyzed existing online discourse, integrating large-scale data collection (329,924 texts), AI-powered classification with expert validation, and network analysis.

The inductive design is necessary as no prior studies have mapped cognitive distortion networks in non-Western languages at population scale; findings are interpreted through CBT theory while remaining agnostic to specific network configurations.

### 2.3 Participants

The corpus comprised 249,414 texts from Russian-speaking individuals discussing psychological topics on publicly accessible online platforms (YouTube psychology channels 85%, forums/social media 15%). Individual demographics were not collected.

### 2.4 Unit of analysis and segmentation strategy

To avoid artifacts arising from arbitrary text segmentation, the single user comment was defined as the indivisible unit of analysis. No internal segmentation (e.g., splitting into sentences or paragraphs) was performed. This decision was theoretically grounded: cognitive distortions (e.g., Rationalization) often manifest across multiple sentences, requiring the full narrative context for accurate detection. Treating the entire comment as a single context window ensures that the co-occurrence of distortions reflects their presence within a specific communicative act, rather than an artifact of aggregation.

### 2.5 Data collection and corpus construction

To ensure the validity of the findings, a purposeful sampling strategy targeting naturalistic psychological discourse was employed. The corpus construction proceeded in three stages: source selection, data acquisition, and filtration pipeline.

#### 2.5.1 Sampling strategy and source selection

Russian-language digital communities were identified across 53 thematic categories related to mental health (see full list in Supplementary material), covering clinical conditions (e.g., Anxiety disorders, Depression, OCD symptoms, and PTSD symptoms), interpersonal challenges (e.g., Family conflicts, Divorce and separation, and Relationship problems), and personal growth topics (e.g., Self-development, Burnout, and Perfectionism). A systematic search on YouTube yielded a candidate list of channels, which were screened against inclusion criteria to ensure high engagement and content relevance:

1. Audience size: Minimum of 2,000 subscribers to exclude inactive communities.
2. Content volume: Minimum of 50 videos per channel to ensure sustained discourse.
3. Reach: Minimum of 300,000 total channel views.
4. Discourse relevance: Manual verification was performed to ensure that the community interactions primarily consisted of autobiographical self-disclosure and personal storytelling, rather than generic reactions or phatic communication.

The final source list comprised 200 channels covering clinical (e.g., OCD, Depression), subclinical (e.g., Perfectionism, Procrastination), and interpersonal (e.g., Divorce, Parenting) domains.

#### 2.5.2 Filtration pipeline and data transformation

The raw dataset underwent rigorous processing to transform unstructured web discourse into an analyzable corpus:

- Technical preprocessing: Input texts were standardized to UTF-8 encoding. Non-textual elements (HTML tags, timestamps, user artifacts) and external links (URLs) were removed to eliminate noise that could interfere with semantic analysis.
- Length constraint: Texts shorter than 200 characters were excluded. This threshold (approx. 10–15 seconds of spoken speech) was empirically selected to filter out reactive comments (e.g., “Great video,” “I agree”). The resulting corpus demonstrated a substantive narrative structure (Mean = 622.3, Median = 435 characters; Range = [200, 8307]), confirming that the selected texts contain sufficient semantic density to manifest complex cognitive distortions.
- Thematic filtering: A keyword-based blocklist was applied to exclude irrelevant domains (political, religious, and spam content).
- Deduplication and dependency control: Exact text duplicates were removed to mitigate the impact of spam bots and repeated posting. Since user identifiers were not retained for ethical reasons (preventing strict user-level nesting), this deduplication step served as the primary control for source dependency.

This multi-stage selection process ensures that the final analytical corpus consists of substantial narratives capable of reflecting cognitive patterns, filtering out low-information noise.

Following automated classification, texts lacking valid distortion annotations ( 4%) were excluded, yielding *N* = 249,414 for analysis.

### 2.6 Classification system

The study classified 18 cognitive distortion types from established CBT frameworks (Beck et al., 1979; Rotter, 1966; Abramson et al., 1978; Festinger, 1954, 1957; Aronson, 1973; Nolen-Hoeksema, 1991; Burns, 1980):

1. All-or-Nothing Thinking
2. Overgeneralization
3. Mental Filter
4. Disqualifying the Positive
5. Fortune Telling
6. Mind Reading
7. Catastrophizing
8. Emotional Reasoning
9. Should Statements
10. Labeling
11. Personalization
12. External Locus of Control
13. Tunnel Vision
14. Social Comparison
15. Rationalization
16. Rumination
17. Learned Helplessness
18. Fairness Fallacy

To address the risk of conflating normative autobiographical self-disclosure (common in social media) with pathological distortions, the system instructions included negative constraints. Specifically for Personalization, the models were instructed to distinguish between simple self-reference (“I felt sad when X happened”) and undue causal attribution (“X happened because I am unworthy”), classifying only the latter as a distortion.

These 18 types were selected through systematic review of CBT literature to maximize theoretical coverage. This enables data-driven identification of natural patterns, testing whether empirically-derived groupings align with existing frameworks (Barriga et al., 2008).

### 2.7 AI classification implementation

The classification was operationalized as a multi-label identification task using pre-trained Large Language Models (LLMs) via API. Following best practices (Demszky et al., 2023), we utilized an ensemble of two models: Qwen2.5:14b (40% of texts) and GPT-4 o1-mini-2024-09-12 (60% of texts). This design aimed to mitigate systematic bias by leveraging orthogonal error profiles (Malberg et al., 2024; Gao et al., 2025). A zero-shot prompting protocol was employed to assign labels directly based on the provided definitions. Fine-tuning was not applied to preserve the models' general semantic reasoning capabilities.

The classification procedure followed two core principles:

1. Explicit evidence requirement: A distortion was coded as present only if the text contained a specific semantic span manifesting the cognitive error, which the models were instructed to extract verbatim. This served as a filter against over-interpretation of ambiguous content.
2. Multi-label logic: Distortions were treated as non-mutually exclusive, allowing a single text to be assigned multiple labels if distinct evidence supported each.

To validate performance, a clinical psychologist independently coded 1,000 texts (stratified random sample) blind to AI classifications. The resulting AI-expert agreement, Cohen's κ = 0.73, indicated substantial reliability, falling within the established range for trained experts (κ = 0.52 − 0.78) (Kuber et al., 2025; Qi et al., 2025). We prioritized this global agreement calculated blindly to prevent researcher bias; consequently, granular per-class statistics were not computed to maintain the blinding protocol and avoid overfitting to the validation set. Full prompt structure is provided in Supplementary material.

To validate the model performance, validation subset was created using stratified random sampling from the full corpus to ensure the representativeness of distortion classes.

Replicability and Open Science. To address the non-deterministic nature of LLMs and ensure exact reproducibility, all API calls were executed with a temperature setting of 0.7 and fixed random seeds 42. The complete classification pipeline is fully documented to support replication. We provide a public repository containing: (1) the verbatim system prompts and few-shot examples used for both models; (2) the Python scripts for text preprocessing, data filtration, and label aggregation; and (3) the code used for network construction, QAP analysis, and bootstrap stability testing.

#### 2.7.1 Construct validity: discourse-cognition mapping

Cognitive distortions are operationalized as propositional reasoning errors detectable through explicit linguistic markers. A distortion is coded when text demonstrates a verifiable logical error (causal misattribution, absolute quantification, and unwarranted certainty) independent of authorial intent or context.

Naturalistic online discourse differs from clinical transcripts: social media employs rhetorical hyperbole, irony, and performative expressions that structurally mimic distortions but lack pathological intent. To address this, the protocol enforced a literal interpretation constraint requiring explicit semantic markers and verbatim textual evidence. Pragmatically ambiguous statements defaulted to null classification.

This conservative design prioritizes precision over recall, intentionally accepting underdetection of implicit distortions to avoid spurious network connections from metaphorical language. Detected patterns represent linguistic markers of cognitive processing styles rather than clinical diagnoses. Representative boundary cases including false positives (idioms misclassified) and false negatives (implicit patterns missed) are documented in Supplementary material.

### 2.8 Network stability and reliability

Split-half reliability was assessed by randomly dividing the dataset into two equal subsamples (*n*_1_ = *n*_2_ = 124, 707 texts) with a fixed random seed (seed = 42) to ensure reproducibility. Split-half reliability was high (QAP *r* = 0.943, *p* < 0.001) based on random division with fixed seed for reproducibility.

Statistical Justification for QAP. Standard statistical tests (e.g., Pearson's correlation) assume that observations are independent. In network analysis, this assumption is violated because edges share nodes (dyadic dependence), which inflates Type I error rates. The Quadratic Assignment Procedure (QAP) overcomes this by treating the entire matrix as the unit of analysis. It computes the correlation between the two split-half networks and compares this observed value against a null distribution generated by randomly permuting the rows and columns of one matrix 5,000 times. A significant QAP result (*p* < 0.001) therefore confirms that the structural pattern of cognitive distortions—specifically, which distortions tend to co-occur with which—is robustly preserved across independent subsamples and is not an artifact of random chance or sample composition.

This high QAP correlation validates the statistical process control (SPC) of our ensemble classification, confirming the process was stable and reproducible across the entire dataset. Following industrial metrology principles, the *N* = 1,000 expert-coded texts served as the process validation sample (calibrating the “machine”), justifying the large-scale (*N* = 249,414) classification.

We computed the Quadratic Assignment Procedure (QAP) correlation between the adjacency matrices of networks constructed independently from each subsample. QAP correlation quantifies network similarity through permutation testing, accounting for the non-independence of network data. Bootstrap stability analysis (*k* = 10, 000 iterations with replacement) was conducted to estimate confidence intervals for network density and clustering coefficient. For each bootstrap sample, we reconstructed the network using identical filtering criteria (count ≥1, lift >1.0). Node stability was operationalized as the proportion of bootstrap samples in which each node appeared.

Network stability was assessed using two thresholds. First, a high-stability threshold (≥95%) was set to identify the “Core Network” of robustly interconnected nodes. Second, a lower “Exploratory Threshold” (≥60%) was used to retain nodes that, while less stable, hold theoretical importance (e.g., Fairness Fallacy, Fortune Telling) and allow for analysis of their peripheral roles. This dual-threshold approach distinguishes the stable network core from less frequent, context-dependent patterns. Nodes appearing in < 60% of samples were excluded.

### 2.9 Network robustness to annotation errors

To address concerns regarding single-annotator reliability, we conducted a sensitivity analysis testing network stability under simulated misclassification scenarios. Annotation errors were modeled by introducing controlled noise into the classification labels: at each noise level (0%–50% in 5% increments), labels were randomly removed (simulating false negatives) and incorrect labels added (simulating false positives) to match the specified error rate.

For each of 11 noise levels, we generated 50 independent noisy datasets (k = 550 total), reconstructed the co-occurrence network using identical filtering criteria (*lift*>1.0, *count*≥1), and computed: (1) network density, (2) clustering coefficient, (3) hub stability (Jaccard similarity between top-5 hubs in noisy vs. original network).

Hub Jaccard ≥0.60 was defined as the stability threshold, indicating preservation of core network structure. This threshold is conservative: values ≥0.60 indicate that at least 3 of 5 primary hubs remain consistent despite annotation errors.

### 2.10 Pairwise co-occurrence analysis

All 153 possible distortion pairs [*C*(18, 2)] were examined for RQ1:

1. Observed frequency (*O*): Count of texts containing both distortions.

2. Expected frequency (*E*):


$$\begin{array}{l} E=\frac{N_{1}\times N_{2}}{N_{\text{total}}} \end{array}$$ (1)

3. Lift ratio:


$$\begin{array}{l} \text{Lift}=\frac{O}{E} \end{array}$$ (2)

indicating association strength. Association rule mining has demonstrated clinical utility in identifying meaningful patterns in healthcare data (Miswan et al., 2021), providing methodological precedent for our approach.

4. Statistical significance: Chi-square tests with Bonferroni correction:


$$\begin{array}{l} \alpha =\frac{0.05}{153}=0.000327 \end{array}$$ (3)

Networks were constructed with filtering criteria: Lift>1.0, minimum co-occurrence count ≥1, *p* < 0.001. Bootstrap stability analysis (*k* = 10, 000) was conducted. Nodes were retained for the final network if they appeared in ≥60% of samples, consistent with our stability threshold. After stability filtering (bootstrap ≥60%), 13 nodes were retained. Of these, 11 demonstrated high stability (≥95%) while two nodes (Fairness 93.0%, Fortune Telling 60.8%) met the inclusion threshold but showed lower stability and were retained for exploratory analysis.

Following established approaches in healthcare data mining, we employed association rule mining to identify cognitive distortion co-occurrence patterns.

### 2.11 Triadic pattern analysis

To address RQ2 (stable triadic configurations), we developed the Integrated Significance Metric (ISM), combining three components: relative frequency (*f*_rel_), average conditional probability (*P*_avg_), and observed-to-expected ratio (OER). The multiplicative formula:

$$\begin{array}{l} \text{ISM}=f_{\text{rel}}\times P_{\text{avg}}\times \text{OER} \end{array}$$ (4)

ensures high values require simultaneous elevation across all components, preventing spurious significance. Here, *f*_rel_ represents relative frequency (observed count divided by total triads), *P*_avg_ is the average conditional probability across all three distortions, and OER is the observed-to-expected ratio under independence.

Analysis covered 816 possible three-way combinations [*C*(18, 3)]. The 99th percentile (ISM = 0.0308) served as the significance threshold, identifying 12 significant triadic patterns.

### 2.12 Software and technical implementation

Data analysis employed Python 3.10 with pandas, scipy, and networkx libraries. Automated classification utilized standard configurations: Qwen2.5:14b and GPT-4 o1-mini-20240912 via API.

## 3 Results

Descriptive Statistics and Distribution. The final analyzed corpus contained a mean of 1.78 distortions per text. Descriptive analysis of the label distribution (see Supplementary Figure S1) revealed that only 4% of the analyzed comments contained zero identified distortions, while the majority of observations contained two or more co-occurring patterns. This prevalence of multiple distortions per single observation justifies the application of network analysis to explore their structural interdependencies.

Cross-Video Robustness. To ensure that the network topology was not contingent on video-specific context or activity levels, a stratified cross-video validation was performed. The dataset, encompassing 10,655 unique video threads, was partitioned into subsets based on interaction density. As detailed in Supplementary Table S4, the structural correlation between high-activity threads and the long tail of sporadic discourse remained consistently high (*r* = 0.75 − 0.80, *p* < 0.001). This invariance indicates that the core network architecture is robust across varying levels of discussion intensity and platform-specific contexts.

Preliminary screening indicated that approximately 4% of the length-filtered initial corpus contained no detectable cognitive distortions; these texts were excluded to focus the analysis on distortion co-occurrence patterns.

Cohen's κ = 0.73 indicated strong agreement between AI and expert classifications. Analysis proceeds hierarchically: individual frequencies establish baseline prevalence, pairwise associations address RQ1 (dyadic co-occurrence), triadic patterns address RQ2 (stable configurations), and network metrics address RQ3 (centrality differences).

The corpus contained 443,447 distortion instances (*M* = 1.78 per text). Most texts contained 1–2 distortions (82.3%), with fewer presenting 3 distortions (12.7% ) or 4+ distortions (5.0%). The distribution demonstrated statistically significant positive skew (*skewness* = 1.97, *p* < 0.001) (Supplementary Figure S1). Supplementary Table S1 presents the frequency distribution of 18 cognitive distortion categories.

### 3.1 RQ1: Dyadic co-occurrence patterns

**Figure 1:**
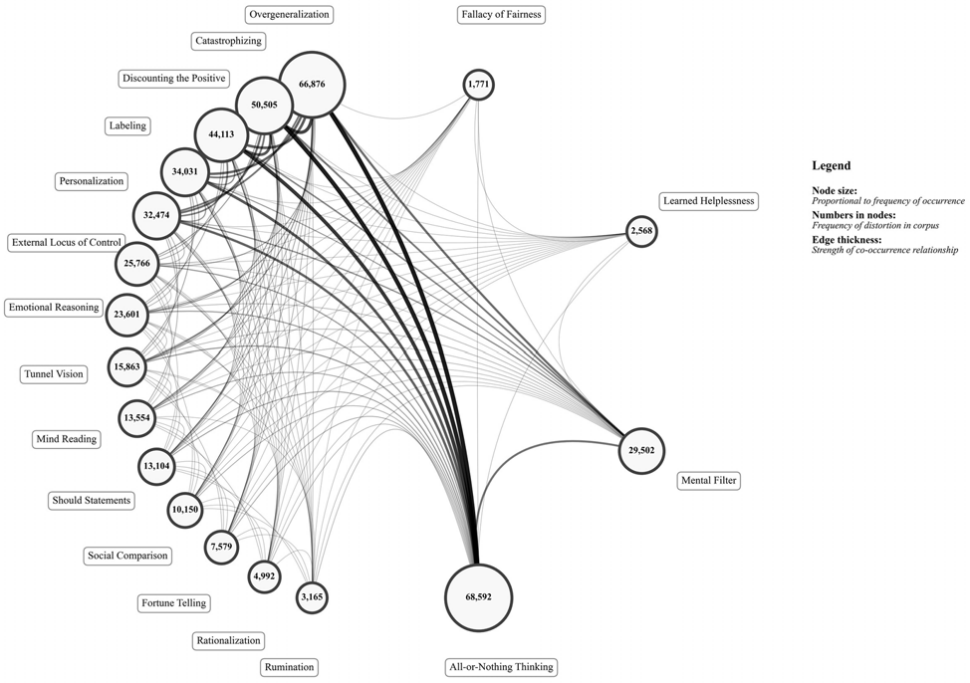
Initial co-occurrence network of all 18 cognitive distortions. Node size is proportional to frequency of occurrence in the corpus; edge thickness represents the strength (Lift Ratio) of the pairwise co-occurrence.

Supplementary Figure S2 presents the complete network structure with gray nodes indicating distortions with zero significant connections after Bonferroni correction. After stability filtering (*bootstrap*≥60%, yielding 13 nodes), 35 significant associations were identified in the final network (*p* < 0.001) :

The initial pairwise co-occurrence analysis identified significant connections among all 18 distortion types. Figure 1 visualizes this complete initial network, where node sizes represent their frequency and edge thickness indicates the strength (Lift Ratio) of co-occurrence prior to stability filtering.

Strongest pairwise associations by lift:

- All-or-Nothing ↔ Catastrophizing: lift = 1.96.
- Catastrophizing ↔ Discounting: lift = 1.88.
- All-or-Nothing ↔ Discounting: lift = 1.87.

### 3.2 RQ2: Triadic configurations

Among the 816 theoretically possible triadic combinations [*C*(18, 3)], 12 distinct patterns exceeded the 99th percentile threshold of *ISM* = 0.0308 (Table 1).

**Table 1:** Statistically significant triadic patterns (99th percentile).

**Rank**	**ISM**	**Frequency**	**Percentage**	**O/E ratio**	**Triadic pattern**
**1**	**0.1282**	**11816**	**4.7%**	**4.9**	**All-or-Nothing + Catastrophizing + Discounting positive**
2	0.1013	12871	5.1%	3.8	All-or-Nothing + Catastrophizing + Overgeneralization
3	0.0759	8084	3.2%	4.6	All-or-Nothing + Catastrophizing +Personalization
4	0.0733	9084	3.6%	3.9	All-or-Nothing + Labeling + Overgeneralization
5	0.0638	9965	4.0%	3.3	All-or-Nothing + Discounting Positive + Overgeneralization
6	0.0476	6223	2.5%	4.0	All-or-Nothing + Discounting Positive + Personalization
7	0.0442	6792	2.7%	3.6	All-or-Nothing + Catastrophizing + Labeling
8	0.0428	7405	2.9%	3.4	Catastrophizing + Discounting Positive + Overgeneralization
9	0.0375	4991	2.0%	4.4	Catastrophizing + Discounting Positive + Personalization
10	0.0358	6569	2.6%	3.0	All-or-Nothing + Personalization + Overgeneralization
11	0.0356	5789	2.3%	3.5	Catastrophizing + Personalization + Overgeneralization
12	0.0308	4278	1.7%	4.2	Catastrophizing + Mental Filter + Overgeneralization

O/E Ratio, Observed/Expected frequency ratio. All triadic patterns exceeded the 99th percentile significance threshold.

### 3.3 RQ3: Global network architecture and stability

The initial analysis identified 35 significant associations (*p* < 0.001) among 13 cognitive distortions that passed the bootstrap stability threshold (appearing in ≥60% of 10,000 samples).

The final network (*N* = 13 nodes, E = 35 edges) demonstrated moderate density (D = 0.449, 95% CI [0.362, 0.582]) and high clustering (C = 0.598, 95% CI [0.485, 0.763]). This indicates that distortions function as an integrated system with local substructures. High architectural reliability was confirmed by split-half analysis (QAP *r* = 0.943, *p* < 0.001).

Node stability analysis (Table 2) confirmed the robustness of the network core:

- Stable core: 11 of the 13 nodes exhibited high stability (≥95% bootstrap appearance), with 10 demonstrating absolute stability (100%) and one (Mind Reading) near-absolute stability (98.8%).
- Peripheral nodes: Two nodes (Fairness Fallacy, 93.0%; and Fortune Telling, 60.8%) were retained for exploratory analysis despite not reaching the 95% high-stability threshold.
- Excluded nodes: Five distortions (Emotional Reasoning, External Locus of Control, Rationalization, Social Comparison, and Learned Helplessness) failed to meet the inclusion threshold (≥60%) and were excluded from the final network analysis.

**Table 2:** Network centrality and bootstrap stability.

**Rank**	**Cognitive distortion**	**Degree**	**Bootstrap stability (%)**
1	Personalization	10	100.0
2	All-or-Nothing Thinking	8	100.0
3	Catastrophizing	8	100.0
4	Discounting Positive	7	100.0
5	Labeling	7	100.0
6	Overgeneralization	7	100.0
7	Mental Filter	6	100.0
8	Rumination	6	100.0
9	Tunnel Vision	6	100.0
10	Mind Reading	2	98.8
11	Should Statements	1	100.0
12	Fairness Fallacy	1	93.0
13	Fortune Telling	1	60.8

### 3.4 RQ3: Network centrality and core structure

Network centrality (Degree) varied significantly, addressing RQ3 (Table 2). Personalization was identified as the network's primary hub (degree = 10). All-or-Nothing Thinking and Catastrophizing also emerged as key nodes (degree = 8 each).

Analysis of this core structure revealed a densely interconnected cluster involving these central nodes as well as Overgeneralization and Discounting. While the strongest association by lift was between All-or-Nothing and Catastrophizing (lift = 1.96, RQ1), the most frequent co-occurrence by raw count was between All-or-Nothing and Overgeneralization (count = 27,845, lift = 1.56).

The analysis also revealed distinct node roles:

- Hubs and triads: A divergence was found between centrality and triadic involvement. While Personalization (degree = 10) was the primary hub, Catastrophizing and All-or-Nothing (degree = 8) dominated complex configurations (appearing in 67% of significant triads). Notably, only 7 of the 18 primary distortions (39%) participated in these stable triadic patterns, suggesting that complex three-way interactions are limited to this specific subset.
- Structural bridge: Mental Filter (degree = 6) showed maximum local clustering (C=1.0) alongside minimal triadic involvement, suggesting it functions as a bridge reinforcing an already-cohesive subgroup (Catastrophizing, Overgeneralization, Discounting).
- Cognitive bridge: Rumination (degree = 6) had a low weighted degree (4.9) but elevated betweenness centrality (0.207), indicating its role in connecting otherwise separate network regions.

**Figure 2:**
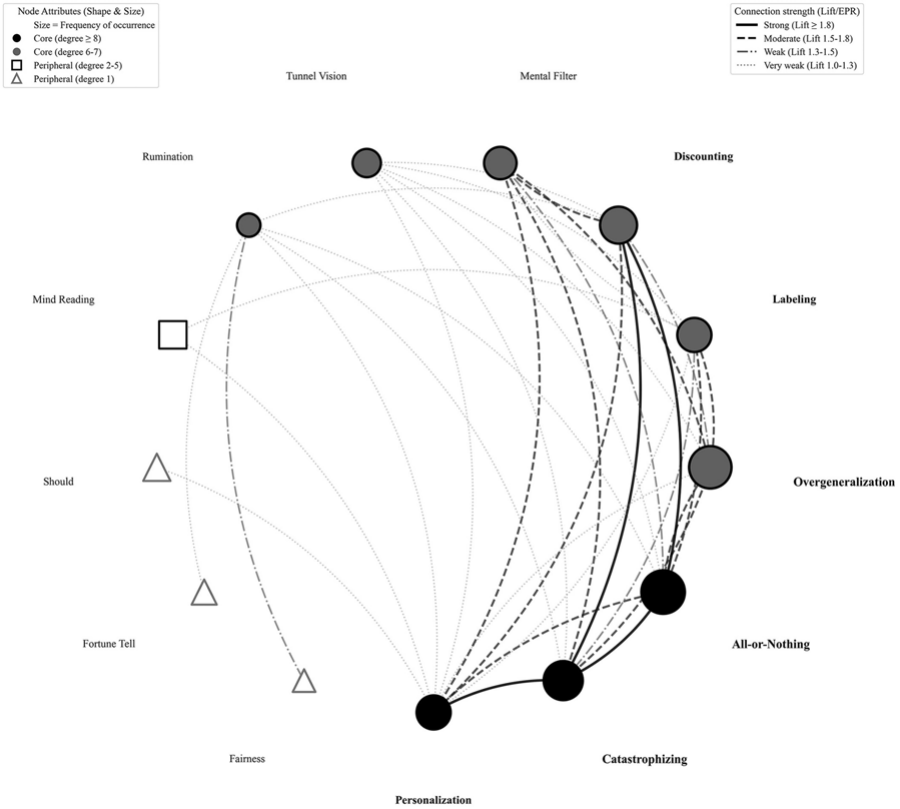
Final core network structure (*N* = 13) after bootstrap validation. This network includes only nodes with high stability (≥60.8%). Node attributes (shape, size) represent centrality (degree), and edge thickness represents association strength (Lift Ratio).

The network exhibited disassortative mixing (r = –0.071), indicating that hub nodes (e.g., Personalization) preferentially connect to less central peripheral nodes. Figure 2 visualizes this final network structure, with node sizes proportional to weighted degree and edge thickness representing Lift Ratio values.

### 3.5 Network robustness under simulated misclassification

To address concerns regarding single-annotator reliability, we conducted a sensitivity analysis testing network stability under simulated misclassification scenarios. Annotation errors were modeled by introducing controlled noise into the classification labels: at each noise level (0%–50% in 5% increments), labels were randomly removed (simulating false negatives) and incorrect labels added (simulating false positives) to match the specified error rate.

For each of 11 noise levels, we generated 50 independent noisy datasets (k = 550 total), reconstructed the co-occurrence network using identical filtering criteria (*lift*>1.0, *count*≥1), and computed: (1) network density, (2) clustering coefficient, and (3) hub stability (Jaccard similarity between top-5 hubs in noisy vs. original network). Hub Jaccard ≥0.60 was defined as the stability threshold.

Network Stability Under Annotation Errors. Sensitivity analysis (Supplementary Table S2) revealed that network architecture remains stable under moderate annotation error but degrades predictably beyond threshold levels. Three distinct stability regimes emerged:

Stable regime (0%–25% noise): Hub structure remained perfectly preserved (Hub Jaccard *M* = 0.968 − 1.000), with minimal variation in network topology. Clustering coefficient showed slight elevation (*M* = 0.515 − 0.849) as random errors introduced spurious triangular connections, but core hub composition remained unchanged. This indicates the network can tolerate misclassification rates up to 25%—well exceeding typical expert disagreement (11%–26% for κ = 0.52 − 0.78)—without structural distortion.

Degrading regime (30%–35% noise): Hub Jaccard dropped sharply to *M* = 0.693 − 0.694, approaching the stability threshold (≥0.60) but remaining structurally coherent and indicating substantial hub turnover. Network density increased (*M* = 0.627) as false positive errors began dominating the signal, creating spurious connections between weakly-associated distortions. However, clustering remained elevated (*M* = 0.866 − 0.867), suggesting local substructures persisted even as global hub hierarchy degraded.

Collapse regime (40%–50% noise): Hub Jaccard collapsed to *M* = 0.340 − 0.454, indicating random hub composition. Paradoxically, density and clustering continued increasing (*M* = 0.732 − 0.793 and *M* = 0.889 − 0.897, respectively) as the network became saturated with false positive connections, approaching a fully-connected random graph. This pattern—rising density concurrent with falling hub stability—is diagnostic of noise-induced structure rather than genuine cognitive architecture.

Interpretation: The network architecture would require systematic misclassification rates exceeding 30%—approximately 2–3 times typical expert disagreement—to show meaningful structural instability (Hub Jaccard < 0.70). The sharp degradation boundary at 30% validates our classification process: if the true error rate approached this threshold, we would observe unstable hub structure in bootstrap analysis (which we do not; 11/13 nodes show ≥95% stability). The observed bootstrap stability thus provides indirect evidence that true classification error is well below 30%, consistent with the measured κ = 0.73 agreement.

Density paradox resolved: The counterintuitive increase in density under noise (from baseline 0.436 to 0.627–0.793) reflects false positive error rather than core-periphery contraction. As annotation quality degrades, random label additions create spurious co-occurrences, artificially inflating connection density. This pattern differs fundamentally from the bootstrap stability results, where density reflects filtering to robust connections. Future research should employ dual-annotator designs to empirically bound error rates and strengthen network validity claims.

Cross-Video Stability. To rule out the influence of video-specific clustering (e.g., dependencies within active discussion threads), a stratified cross-video validation was performed on 10,655 distinct video sources. The dataset was partitioned into “high-activity” and “low-activity” subsets using three data-driven thresholds (median, Pareto top-20%, and top-quartile). As shown in Supplementary Table S4, the network topology remained highly stable across all stratifications, with QAP correlations ranging from *r* = 0.751 to *r* = 0.803 (*p* < 0.001). The strong structural correspondence (*r*≈0.79 on average) indicates that approximately 62% of the network variance is shared between the dense video communities and the sporadic long tail, confirming that the core associations are robust to video-specific dynamics.

## 4 Discussion

### 4.1 Theoretical implications: a core and periphery model

This exploratory investigation revealed a hierarchically organized cognitive distortion network in Russian discourse, structured around a stable core (11 nodes) and less stable periphery (2 nodes). Unexpectedly, Personalization emerged as the primary hub despite collectivistic attribution patterns typically reducing self-focus. This pattern discovery supports network-based models while highlighting the need for cross-cultural replication to distinguish universal architecture from cultural contingency.

The finding that Personalization emerged as the network's primary hub (degree = 10) aligns with network theories suggesting high-degree nodes are optimal treatment targets (Castro et al., 2024). Its central role, as identified in our results, suggests it may function as a key vulnerability that activates other distortions. However, we cannot determine whether this centrality reflects universal cognitive mechanisms or Russian cultural attribution patterns without cross-cultural replication.

Personalization's centrality (degree = 10) aligns with transdiagnostic models emphasizing shared mechanisms across diagnostic boundaries (Norton and Paulus, 2017; Sloan et al., 2017).

The co-dominance of All-or-Nothing and Catastrophizing in both pairwise (lift = 1.96) and triadic patterns (67% presence) confirms their role as a cognitive core. This empirically supports Beck's theory of dichotomous thinking as a fundamental vulnerability and aligns with clinical findings linking this triad to comorbid anxiety and depression (Tanıgör et al., 2025). The frequent co-occurrence with Overgeneralization (count = 27,845) further suggests these distortions share common activation pathways.

In contrast, nodes like Fortune Telling (60.8% stability, degree = 1) and Fairness Fallacy (93.0%, degree = 1) were confirmed to be peripheral. Their low stability and connectivity—a key finding of this study—suggest they are not central drivers of the system but rather context-dependent or secondary distortions. This supports the utility of our dual-threshold stability analysis in distinguishing core architecture from node-specific noise.

Mental Filter presented a unique profile. Despite moderate connectivity, its maximum local clustering (C = 1.0) and minimal triadic involvement suggest it functions as a structural bridge, reinforcing existing negative patterns (like Catastrophizing and Overgeneralization) rather than driving new ones. Therapeutically, this implies addressing its upstream drivers (e.g., All-or-Nothing) may be more effective than challenging the filter itself.

### 4.2 Implications for cognitive theory

The structural properties of the observed network suggest several theoretical propositions regarding the architecture of cognitive distortions in naturalistic discourse:

**1. The potential foundational role of binary thinking**. The status of “All-or-Nothing Thinking” as a central hub is consistent with CBT accounts suggesting that dichotomous reasoning may function as an early-stage cognitive simplification mechanism. This centrality in naturalistic discourse suggests it could serve as a frequently activated heuristic that facilitates the emergence of subsequent distortions, though experimental validation would be required to establish causal directionality.

**2. Potential cognitive amplification patterns**. The identification of strong triadic clusters, such as the Catastrophizing—Fortune Telling—Should Statements triad, suggests the possibility of mutually reinforcing cognitive patterns. In this configuration, the prediction of a negative outcome (Fortune Telling) appears structurally linked to an exaggeration of its perceived severity (Catastrophizing) and rigid normative demands (Should Statements). Whether these patterns constitute self-sustaining cycles that amplify psychological distress remains an empirical question for future research.

**3. Structural similarity across contexts**. The high stability of these patterns across 10,655 distinct video sources suggests that the structural organization of cognitive distortions may reflect general features of information processing under stress. This finding raises the question of whether similar network architectures might be observed in clinical populations, warranting direct comparison studies to identify both commonalities and potentially distinctive clinical features.

### 4.3 Clinical implications

The identification of stable network structures in naturalistic discourse has several potential implications for clinical practice and intervention design:

#### 4.3.1 Targeting high-centrality nodes

If the hub status of “All-or-Nothing Thinking” observed in online discourse reflects a similar architecture in clinical presentations, therapeutic interventions that specifically address dichotomous reasoning early in treatment may have cascading effects on interconnected distortions. This would suggest prioritizing cognitive restructuring techniques focused on identifying false dichotomies and exploring middle-ground alternatives.

#### 4.3.2 Addressing distortion clusters

The presence of tight triadic clusters (e.g., Catastrophizing—Fortune Telling—Should Statements) suggests that certain cognitive distortions may co-activate as functionally integrated units. Clinicians might benefit from anticipating these co-occurrence patterns: when a client exhibits catastrophic thinking about future events, proactive exploration of underlying rigid “should” statements and negative predictions may be warranted, even if not spontaneously reported.

#### 4.3.3 Assessment considerations

While experienced clinicians informally recognize that certain cognitive distortions tend to co-occur, formalized assessment protocols (e.g., CDS, ATQ, thought records) typically evaluate each distortion independently, generating separate severity scores without systematically examining co-activation patterns. Our corpus analysis reveals that distortions co-occur at a mean rate of 1.78 per text segment (minimum 200 characters, approximately 2 min of naturalistic speech), suggesting they function as interconnected systems.

This finding points to a potential enhancement of current practice: systematizing the informal pattern recognition that experienced clinicians already employ. Rather than relying solely on separate distortion scores, intake procedures could incorporate network-informed screening that explicitly maps characteristic co-occurrence signatures. For instance, when catastrophizing is detected, protocols could prompt systematic assessment of its empirically-identified cluster partners (all-or-nothing thinking, should statements), even when not spontaneously reported. This formalization of clinical intuition could improve case conceptualization consistency, particularly for less experienced practitioners.

#### 4.3.4 Measurement-based care

The stability of network architecture across diverse contexts suggests that repeated network assessments during treatment could serve as a progress indicator. Changes in network density or hub centrality might reflect meaningful shifts in cognitive processing, offering a quantitative complement to traditional symptom measures.

#### 4.3.5 Caution

These implications remain tentative pending validation in clinical samples. The current findings describe discourse-level co-occurrence patterns in non-clinical online contexts and cannot be assumed to directly map onto cognitive-mechanistic processes in therapeutic settings. Controlled studies comparing network structures across clinical and non-clinical populations, and tracking network changes during evidence-based interventions, are necessary next steps.

### 4.4 Methodological strengths: human-equivalence and emergent stability

Methodologically, this study establishes two key principles for large-scale digital psychology. First, our classification process achieved human-equivalent reliability. As noted, our AI-expert agreement (κ = 0.73) aligns with published benchmarks for human inter-rater reliability on the same complex task (Kuber et al., 2025; Qi et al., 2025). This suggests our process operates at the human “gold standard” level, where the limiting factor is the inherent ambiguity of the task, not a model deficiency (Jia et al., 2025).

Second, we demonstrate a clear case of emergent system stability. The final network's architectural robustness (Bootstrap stability ≥95% for 11 core nodes) significantly exceeds the reliability of its individual components (classification κ = 0.73). This +22 percentage-point gap confirms that the macro-level network structure is a robust, emergent property of the data, highly insensitive to micro-level classification “noise” or random error. This finding, combined with the high reproducibility (QAP *r* = 0.943), provides empirical proof that the detected architecture is a genuine and stable phenomenon.

### 4.5 Cultural context and interpretive ambiguity

The centrality of Personalization presents a theoretical puzzle. Cross-cultural meta-analyses indicate that collectivistic cultures typically show external attribution patterns (Oyserman et al., 2002), yet Russia's moderately collectivistic orientation would predict situational rather than self-focused attribution. The observed prominence of Personalization may therefore reflect: (a) context-specific patterns in online mental health discourse and platform-driven self-disclosure; (b) bias within the Western CBT framework regarding distortion classification; or (c) genuine psychopathological processes distinct from normative cultural attribution. Cross-cultural replication is essential to resolve this interpretive ambiguity.

### 4.6 Theoretical implications

The identified network stability raises the fundamental question of whether these co-occurrence patterns reflect underlying cognitive architecture or specific discursive norms. From a cognitive perspective, the centrality of Personalization supports theoretical models where self-referential attribution errors function as “gateway” distortions that activate secondary processing biases like Catastrophizing. Alternatively, these configurations may reflect cultural narrative templates or platform affordances that favor specific modes of emotional expression. A mechanism of reciprocal reinforcement is proposed, wherein latent cognitive vulnerabilities are amplified and stabilized by socially available linguistic scripts. Consequently, the observed core-periphery structure suggests that cognitive distortions operate as a hierarchical system rather than independent errors, though establishing strict causal directionality requires future longitudinal validation.

### 4.7 Generative mechanisms: cognitive dynamics vs. discursive reinforcement

The observed co-occurrence patterns raise questions regarding their generative mechanisms. Cross-sectional data cannot distinguish between three non-exclusive processes: (1) sequential activation (one distortion triggers another); (2) concurrent activation (simultaneous processing); and (3) discursive reinforcement (socially learned templates).

Cognitive theories propose that distortions may activate each other through spreading activation in associative networks (Beck et al., 1979), while sociocultural perspectives emphasize that certain cognitive patterns may reflect culturally available linguistic scripts rather than individual pathology. The robust stability of these co-occurrence patterns suggests a mechanism of **reciprocal reinforcement**, where internal cognitive processes and external discursive norms mutually stabilize each other (Sloan et al., 2017). Rather than operating in isolation, cognitive vulnerabilities (e.g., the logical link between All-or-Nothing and Catastrophizing) are likely reinforced and amplified by culturally available narrative templates.

To disentangle these layers empirically, future research requires a differential analytical strategy. First, to distinguish cognitive pathology from social learning (Mechanism 3), researchers can analyze deviations from the group norm. Patterns that are universal across the entire corpus likely reflect shared linguistic norms (social templates), whereas patterns unique to specific individuals (individual variance) reflect personal cognitive dynamics (Harris et al., 2025). Second, for these individual-specific patterns, intra-textual narrative analysis of longer texts (>400 characters) can map the temporal flow of distortions, distinguishing antecedent cognitive drivers from consequent reactions. Such idiographic network approaches, combined with within-text sequential analysis, could reveal whether distortions operate through sequential cascades or concurrent activation mechanisms.

## 5 Limitations

Regarding content effects, the dominance of YouTube comments (85%) introduces a platform-specific discourse style characterized by high autobiographical self-disclosure. While our classification prompts explicitly differentiated between self-reference and Personalization, the high centrality of Personalization may partially reflect the “diary-like” norms of the platform, where users are primed to relate content to their own experiences. Future research should validate these patterns in more neutral contexts to isolate the “medium effect” from the “cognitive effect.”

The Russian-language focus limits cross-cultural generalizability. We cannot determine whether Personalization's centrality reflects universal cognitive mechanisms or Russian cultural attribution patterns. Cross-cultural replication is required to test pattern universality.

Two nodes, while meeting the inclusion threshold (≥60%), demonstrated lower stability compared to the network core: Fortune Telling (60.8%) and Fairness Fallacy (93.0%), falling just short of the high-stability (95%) mark. Fortune Telling's low bootstrap appearance rate may reflect genuine instability in its co-occurrence patterns, context-dependent activation, or insufficient base rate in the dataset. Fairness Fallacy's marginal stability (93.0%) suggests it may reflect sampling variability rather than fundamental instability. Both nodes were retained as exploratory findings due to theoretical importance in CBT frameworks, but their network positions warrant cautious interpretation pending replication.

While both were retained due to theoretical importance, their network positions warrant cautious interpretation. The low stability of Fortune Telling may reflect: (1) genuine instability in its co-occurrence patterns, suggesting it activates more variably across contexts; (2) lower base rate in the dataset, reducing statistical power for detecting stable associations; or (3) measurement sensitivity, whereby Fortune Telling overlaps conceptually with other distortions (e.g., Catastrophizing). Five distortions (Emotional Reasoning, External Locus of Control, Rationalization, Social Comparison, and Learned Helplessness) did not meet inclusion criteria for the final network (appearing in < 60 % of bootstrap samples) and were excluded from the analysis.

Two AI models (Qwen2.5:14b, GPT-4 o1-mini-20240912) were used for text classification on different subsets. This was a deliberate ensemble design intended to enhance reliability and implement fault tolerance. Rather than relying on a single, potentially biased model, we employed two architecturally distinct models to leverage orthogonal error profiles (Malberg et al., 2024; Gao et al., 2025). This design strategically converts systematic bias (a high risk in single-model architectures) into random, annullable error, which is mitigated by the large sample size.

The validity of this ensemble process was confirmed not by inter-model consistency (which is not the goal of an orthogonal ensemble), but by two superior metrics: (1) its human-equivalent reliability (κ = 0.73) when benchmarked against an expert (Qi et al., 2025; Kuber et al., 2025), and (2) the emergent stability (≥95%) and reproducibility (QAP *r* = 0.943) of the final network. Therefore, while direct inter-model consistency (LLM-vs.-LLM) could be measured, it is not considered the primary validation metric in this orthogonal ensemble design. The validity of our approach is instead demonstrated by the proven human-equivalence of the *process* and the robustness of the product.

Regarding the validation of the computational classifier, manual verification was conducted by a single expert rater, precluding calculation of inter-rater reliability. However, sensitivity analysis demonstrated that the network structure remains stable under 30% simulated annotation error—far exceeding realistic expert disagreement rates (11%–26% for κ = 0.52–0.78). The macro-level network architecture exhibits emergent stability significantly exceeding component-level classification reliability (κ = 0.73), confirmed by convergent validation (split-half QAP r = 0.943, bootstrap ≥95% ). Future studies should employ multiple independent raters to establish robust ground truth labels.

Cross-sectional design prevents causal inferences. Self-selection bias and platform-specific factors limit clinical generalizability. AI classification (κ = 0.73) may miss context-dependent meanings or cultural nuances. Russian-language focus limits cross-linguistic generalizability. Absence of demographic data limits our ability to examine age, gender, or clinical status as moderators. However, the large sample size (*N* = 249,414) and demographic diversity of public YouTube psychology channels suggest reasonable heterogeneity. Future studies should incorporate demographic controls to test pattern stability across subgroups.

Replication in independent samples is needed before drawing firm conclusions about pattern universality.

Finally, the dataset spans a volatile period (2020–2024), encompassing significant global and regional events. We did not stratify analyses by year or specific platform mechanics. Future research should investigate temporal dynamics to determine if the identified macro-level network stability persists across varying socio-historical contexts.

## 6 Conclusion and future work

This study explored three research questions regarding cognitive distortion co-occurrence patterns in naturalistic Russian-language discourse. Bootstrap stability filtering (k = 10,000, ≥60% threshold) identified a robust network of 13 cognitive distortions with 35 significant associations (density = 0.449, clustering = 0.598), demonstrating moderate-to-high interconnectedness. Split-half reliability confirmed architectural stability across independent sub-samples.

**RQ1 (Dyadic co-occurrence):** The analysis confirmed that distortions co-occur selectively rather than randomly, with 35 robust associations identified after rigorous correction and validation. Theoretically related pairs (such as All-or-Nothing/Catastrophizing) demonstrated the strongest connections, consistent with established cognitive models.

**RQ2 (Triadic configurations):** The study identified a core set of 12 stable triadic configurations, suggesting that complex interactions are limited to a specific subset of distortions. The dominance of triads involving All-or-Nothing, Catastrophizing, and Discounting aligns with Beck's theory, highlighting a foundational cognitive vulnerability pattern. The stability of these patterns was confirmed via bootstrap resampling.

**RQ3 (Network centrality):** Centrality varied significantly across distortions, confirming that certain distortions function as core nodes. Personalization was identified as the network's primary hub, with All-or-Nothing and Catastrophizing also demonstrating high connectivity. This heterogeneity, combined with the five distortions that showed no significant connections, supports a network approach over independent-symptom frameworks. Eleven of thirteen nodes demonstrated high stability (≥95%), with ten showing absolute stability (100%). Two nodes—Fairness Fallacy (93.0%) and Fortune Telling (60.8%)—exhibited lower stability, suggesting context-dependent activation or insufficient base rates for robust detection.

The convergence of high split-half reliability and bootstrap node stability (10 of 13 nodes at 100%) confirms the identified network represents a replicable cognitive architecture in Russian-language discourse rather than sampling artifact, though cross-cultural replication is needed to test universality. The moderate density (0.449) and elevated clustering coefficient (0.598) indicate distortions operate as an integrated system with localized substructures, supporting network models of psychopathology over independent-symptom frameworks. The strong co-occurrence of All-or-Nothing thinking with both Catastrophizing (lift = 1.96) and Overgeneralization (count = 27,845) aligns with cognitive-behavioral models proposing cascading effects in dysfunctional thinking, where dichotomous evaluations trigger catastrophic interpretations and broad negative generalizations.

This study establishes the feasibility of population-scale cognitive pattern analysis and documents network architecture in Russian discourse. If patterns replicate cross-culturally, findings suggest cluster-based interventions targeting hub nodes (Personalization, All-or-Nothing) may prove effective. However, clinical implications remain preliminary pending validation studies. The convergence of split-half reliability and bootstrap stability indicates a genuine cognitive phenomenon worth systematic investigation. Future research must test whether these structural patterns predict treatment outcomes and generalize beyond Russian online discourse.

Methodologically, this research demonstrates the feasibility of large-scale automated pattern detection (Cohen's κ = 0.73) combined with rigorous stability assessment. The bootstrap approach (k = 10,000) and split-half validation provide replicable standards for distinguishing robust patterns from statistical noise in naturalistic language data. Future research should replicate these patterns in independent samples and clinical populations to establish generalizability beyond Russian-language online discourse.

Expansion to 1.8M comments would enable detection of weaker connections and temporal patterns. Cross-cultural replication is critical: parallel analysis of equivalent corpora in diverse languages (English, Mandarin, Arabic, and Spanish) would test whether Personalization's centrality reflects universal cognitive mechanisms or Russian-specific cultural attribution patterns. Clinical validation studies are needed to verify whether these network patterns predict treatment outcomes and inform intervention strategies. Idiographic network approaches could extend this work to personalized treatment planning, while integration with LLM-based therapeutic systems may enable real-time cognitive distortion detection and intervention.

Finally, our study provides strong evidence for the existence of a highly stable network. Future research must test its universality. It remains an open question whether this topology is a fundamental “attractor” in the data or one of several stable states dependent on the LLM 'observer.' Sensitivity analyses using different model families are needed to distinguish invariant network properties from measurement artifacts.

Exploratory Design Implications. As a discovery-oriented study, findings are hypothesis-generating rather than hypothesis-confirming. The identified architecture represents a replicable empirical pattern (QAP r = 0.943, bootstrap stability ≥95%) but cannot establish causality or universality. Key constraints: (1) cross-sectional design precludes temporal dynamics; (2) single-language sample limits cross-cultural generalization; and (3) patterns may reflect platform-specific discourse. Confirmatory replication in independent Russian and cross-linguistic samples is essential before drawing clinical implications. Current findings warrant targeted hypothesis testing, not immediate application.

## Data Availability

The original contributions presented in the study are included in the article/Supplementary material, further inquiries can be directed to the corresponding author.
