# Supplementary material for: Validating the architecture of cognitive distortions in Russian discourse using artificial intelligence and bootstrap analysis
Source: Front Psychol. 2026 Feb 23;17:1740864. doi: 10.3389/fpsyg.2026.1740864 (PMC12968237; doi:10.3389/fpsyg.2026.1740864)
Supplement: Supplementary file 1 [file Data_Sheet_1.pdf]

Supplementary Material

1 COGNITIVE DISTORTION ANALYSIS PROTOCOL

1.1 LLM Prompt. Russian Original Version

Проанализируйте следующий текст с позиции психолога-практика в современном КПТ подходе.

Идентифицируйте и классифицируйте информацию согласно указанным категориям. Используйте строго указанные теги для каждой категории ответа.

Что нужно сделать:

1. Определите все когнитивные искажения в тексте клиента

2. Определите пригодность фрагментов для использования в научных данных. Нужны исключительно истории людей про себя, развернутые мнения по вопросам. Спам, религия, шутки и политика исключи.

Структура ответа: Ответы указывать по каждому пункту ИСКЛЮЧИТЕЛЬНО в тегах с <>

1. <cognitive_distortions> Перечислите все когнитивные искажения, которые присутствуют в тексте. Для каждого искажения: • Название искажений через запятую, строго в формулировке из списка ниже. Своё придумывать запрещаю • Конкретная цитата из текста, иллюстрирующая данное искажение

Полный каталог когнитивных искажений в КПТ:

Катастрофизация — преувеличение негативных последствий событий Избегать: Не путать с реалистичной оценкой серьёзных проблем или обоснованной тревогой о действительно значимых рисках.

Чрезмерное обобщение — распространение выводов из одного случая на все ситуации Избегать: Не путать с описанием повторяющихся паттернов поведения, если человек приводит несколько примеров, подтверждающих закономерность.

Дихотомическое (чёрно-белое) мышление — восприятие ситуаций в крайних категориях Избегать: Не путать с чёткими категориями там, где они уместны (например, «это законно/незаконно») или с эмоциональным усилением для выразительности.

Персонализация — принятие чрезмерной ответственности за внешние события Избегать: Идентифицировать только когда автор винит себя за то, на что реально не мог повлиять (играет роль всемогущего). Задайте вопрос: это действительно было в его власти? Личные истории о собственных переживаниях и реакциях — не персонализация.

Мысленный фильтр — избирательное внимание к негативным деталям Избегать: Не путать с фокусом на проблеме в контексте запроса помощи или обсуждения конкретной трудности.

Навешивание ярлыков — использование негативных ярлыков вместо описания конкретного поведения Избегать: Не путать с самоидентификацией («я интроверт», «я перфекционист») или с использованием общепринятых терминов для описания состояний.

Обесценивание позитивного — игнорирование положительных аспектов жизни Избегать: Не путать с честным признанием, что позитивные моменты не компенсируют текущую боль, или с фокусом на проблеме в момент обращения за помощью.

Мышление жертвы — восприятие себя как пассивной жертвы обстоятельств Избегать: Не путать с описанием реального опыта виктимизации, травмы или объективно ограниченного контроля над ситуацией (болезнь, насилие, дискриминация).

Эмоциональное обоснование — принятие эмоций как доказательства истинности суждений Избегать: Не путать с описанием эмоций как субъективного опыта («я чувствую себя одиноким») или с эмоциональной валидацией собственных переживаний.

Долженствование — жёсткие требования к себе и другим Избегать: Не путать с выражением ценностей, предпочтений или разумных ожиданий («хотелось бы», «было бы хорошо»).

Предсказание будущего — уверенность в негативном исходе событий Избегать: Не путать с реалистичным прогнозом на основе опыта или с выражением беспокойства о возможном развитии событий («боюсь, что», «переживаю, что может»).

Ментальная фильтрация — избирательное внимание к негативу Избегать: То же, что и «Мысленный фильтр» (возможно, дублирование в вашем списке — рекомендую оставить один термин).

Туннельное мышление — фокус только на негативных аспектах ситуации Избегать: Не путать с обсуждением конкретной проблемы, требующей решения, или с временным эмоциональным состоянием.

Рационализация — поиск "разумных" объяснений для иррациональных страхов Избегать: Не путать с попытками понять причины своих чувств или с рефлексией над опытом.

Чтение мыслей — убеждённость в знании мыслей других без доказательств Избегать: Не путать с интерпретацией очевидных сигналов (мимика, слова, действия) или с описанием своих впечатлений («мне показалось», «у меня возникло ощущение»).

Руминация — постоянное "пережёвывание" негативных мыслей Избегать: Не путать с разовым описанием проблемы или с попыткой осмыслить сложную ситуацию. Руминация предполагает циклическое, навязчивое возвращение к одним и тем же мыслям.

Сравнение — постоянное сравнение себя с другими с негативным исходом Избегать: Не путать с единичным упоминанием сравнения для иллюстрации или с нейтральным/позитивным сравнением.

Иллюзия справедливости — ожидание, что мир должен быть справедливым Избегать: Не путать с выражением несогласия с несправедливостью или с описанием морального дискомфорта от несправедливой ситуации.

Выученная беспомощность — убеждение в невозможности повлиять на ситуацию Избегать: Не путать с признанием реальных ограничений, временной деморализацией или с просьбой о помощи в ситуации, где ресурсы действительно исчерпаны.

</cognitive_distortions>

2. <content_utilization> • В начале поставить цифру 0 если это не подходит для данных. 1 если подходит. • Фрагменты, подходящие для иллюстративных историй, полностью </content_utilization>

ВАЖНО отвечать только на эти 2 пункта в соответствующих тегах. Указаны критерии наполнения пунктов

Анализируемый комментарий: CONTENT

1.2 LLM Prompt. English Translation

Analyze the following text from the perspective of a practicing psychologist using a modern CBT approach. Identify and classify information according to the specified categories. Use strictly designated tags for each response category.

1. Identify all cognitive distortions in the client's text.

2. Determine the suitability of fragments for use in scientific data. Only personal stories, detailed opinions on issues are needed. Exclude spam, religion, jokes, and politics.

Response structure: Provide responses for each point EXCLUSIVELY in tags with <>

1. <cognitive_distortions>

List all cognitive distortions present in the text.

For each distortion: Name of distortions separated by commas, strictly in the wording from the list below. Creating your own is prohibited. Specific quote from the text illustrating this distortion.

Complete catalog of cognitive distortions in CBT:

All-or-Nothing Thinking — perception of situations in extreme categories. Avoid: Do not confuse with clear categories where appropriate (e.g., "this is legal/illegal") or with emotional intensification for expressiveness.

Overgeneralization — extending conclusions from one case to all situations. Avoid: Do not confuse with description of recurring behavioral patterns if person provides several examples confirming the pattern.

Mental Filter — selective attention to negative details. Avoid: Do not confuse with focus on problem in context of help request or discussion of specific difficulty.

Disqualifying the Positive — ignoring positive aspects of life. Avoid: Do not confuse with honest acknowledgment that positive moments do not compensate for current pain, or with focus on problem when seeking help.

Fortune Telling — certainty in negative outcome of events. Avoid: Do not confuse with realistic forecast based on experience or with expression of concern about possible development of events ("afraid that," "worry that might").

Mind Reading — conviction in knowing thoughts of others without evidence. Avoid: Do not confuse with interpretation of obvious signals (facial expressions, words, actions) or with description of own impressions ("seemed to me," "got the feeling").

Catastrophizing — exaggeration of negative consequences of events. Avoid: Do not confuse with realistic assessment of serious problems or justified anxiety about truly significant risks.

Emotional Reasoning — accepting emotions as proof of truth of judgments. Avoid: Do not confuse with description of emotions as subjective experience ("I feel lonely") or with emotional validation of own experiences.

Should Statements — rigid demands on self and others. Avoid: Do not confuse with expression of values, preferences, or reasonable expectations ("would like," "would be good").

Labeling — using negative labels instead of describing specific behavior. Avoid: Do not confuse with self-identification ("I'm an introvert," "I'm a perfectionist") or with use of commonly accepted terms for describing states.

Personalization — taking excessive responsibility for external events. Avoid: Identify only when author blames themselves for what they realistically could not influence (playing omnipotent role). Ask: was this truly within their control? Personal stories about own experiences and reactions are not personalization.

External Locus of Control — perceiving oneself as passive victim of circumstances. Avoid: Do not confuse with description of real victimization experience, trauma, or objectively limited control over situation (illness, violence, discrimination).

Tunnel Vision — focus only on negative aspects of situation. Avoid: Do not confuse with discussion of specific problem requiring solution, or with temporary emotional state.

Social Comparison — constant comparison of self with others with negative outcome. Avoid: Do not confuse with single mention of comparison for illustration or with neutral/positive comparison.

Rationalization — searching for "reasonable" explanations for irrational fears. Avoid: Do not confuse with attempts to understand causes of one's feelings or with reflection on experience.

Rumination — constant "chewing over" of negative thoughts. Avoid: Do not confuse with one-time description of problem or with attempt to comprehend complex situation. Rumination implies cyclical, obsessive return to same thoughts.

Learned Helplessness — belief in impossibility of influencing situation. Avoid: Do not confuse with acknowledgment of real limitations, temporary demoralization, or with request for help in situation where resources are truly exhausted.

Fairness Fallacy — expectation that world should be fair. Avoid: Do not confuse with expression of disagreement with injustice or with description of moral discomfort from unjust situation.

2. <content_utilization> At the beginning, put digit 0 if unsuitable for data. 1 if suitable. Fragments suitable for illustrative stories, in full.

IMPORTANT: Answer only these 2 points in corresponding tags. Criteria for filling points are specified.

Comment for analysis:

{CONTENT}

1.3 Thematic Categories for Data Collection

Below is the numbered list of 53 thematic categories used for the purposeful sampling of YouTube channels and online communities. These categories cover a broad spectrum of clinical, subclinical, and interpersonal psychological issues.

1. Stress and stress management
2. Anxiety disorders
3. Depression
4. Relationship problems
5. Self-esteem issues
6. Finding life purpose
7. Family conflicts
8. Workplace difficulties
9. Burnout
10. Decision making
11. Grief and loss
12. Phobias and fears
13. Loneliness
14. Motivation problems
15. Self-development and personal growth
16. Anger management
17. Social anxiety
18. Panic attacks
19. Adaptation to change
20. Sleep problems
21. Overcoming past experiences
22. Addictions
23. Parenting difficulties
24. Perfectionism
25. Sexual problems
26. Midlife crisis
27. Emotional abuse
28. Trust issues
29. Communication difficulties
30. Inferiority complex
31. Comorbid conditions (e.g., chronic illness and mental health)
32. Eating problems
33. Time management
34. Creative crisis

35. Personality disorders
36. Psychosomatic problems
37. Bulimia and anorexia
38. PTSD symptoms (Post-Traumatic Stress Disorder)
39. OCD symptoms (Obsessive-Compulsive Disorder)
40. Tension relief problems
41. Existential crises
42. Victimhood and submissiveness
43. Excessive control
44. Changing old behavior patterns
45. Guilt and shame
46. Adolescent adaptation problems
47. Individuality and self-expression problems
48. Difficulties in achieving goals
49. Divorce and separation
50. Goal setting
51. General Psychology (services like Yasno)
52. Coaching
53. Life difficulties in relationships (user stories)

2 QUALITATIVE ANALYSIS OF CLASSIFICATION BOUNDARIES

To ensure construct validity, we analyzed boundary cases where algorithmic logic interacts with pragmatic ambiguity. Below are representative examples illustrating True Positives, True Negatives, False Positives, and False Negatives. All examples are drawn from the final dataset (text length > 200 characters).

2.1 True Positives (Correct Detection)

Case 1: Personalization and All-or-Nothing Thinking

Text (Russian): В детстве я раздавил улиточку у нее на глазах. Безумно стыдно за этот поступок. Мне никогда не смыть вину за убитых и замученных малышей. Боюсь меня это никогда не отпустит, сколько бы животных я не спас, мне никогда не смыть вину.

English Translation: In childhood I crushed a snail in front of her eyes. I am insanely ashamed of this act. I will never wash away the guilt for the killed and tortured little ones. I fear this will never let me go, no matter how many animals I save, I will never wash away the guilt.

- AI Classification: Personalization, All-or-Nothing Thinking
- Expert Annotation: Personalization, All-or-Nothing Thinking

The text demonstrates Personalization through the pathological extension of responsibility: the user attributes adult-level moral culpability to a developmental stage of childhood, defining their identity as a "degenerate" rather than viewing the act as a behavioral error. All-or-Nothing Thinking

is confirmed by absolute quantifiers ("never wash away," "no matter how many"), which cognitively block the possibility of redemption despite subsequent positive actions.

Case 2: Labeling and Overgeneralization

Text (Russian): Эйчары - полные кретины, получающие зп за непрофессионализм. Потому что самые преданные и добросовестные работники это люди под 50. А мои ровесники слишком уважают себя. А взрослые не бухтят на работе, фигачат в полную меру.

English Translation: HRs are complete cretins getting paid for unprofessionalism. Because the most devoted and conscientious workers are people under 50. My peers respect themselves too much. But adults do not grumble at work, they work to the fullest.

- AI Classification: Labeling, Overgeneralization
- Expert Annotation: Labeling, Overgeneralization

The user assigns a global negative label to a professional group, fitting the definition of Labeling. The statement also extends characteristics to entire age cohorts without exception, which fits Overgeneralization. The model correctly flagged these distortion patterns.

2.2 True Negatives (Correct Rejection)

Case 3: Justified Emotion vs. Emotional Reasoning

Text (Russian): Зашла в класс, учитель ударил меня ладонью в нос. У меня пошла кровь. Теперь я испытываю злость и ненавижу того учителя, хотя в школе он мне очень нравился. А случившееся меня обескуражило. До сих пор не понимаю, как такое можно было допустить.

English Translation: I entered the class, the teacher struck me in the nose with his palm. I started bleeding. Now I feel anger and hate that teacher, although I liked him before. The incident discouraged me. I still dont understand how this could have been allowed.

- AI Classification: None
- Expert Annotation: None

The text contains strong negative emotions (hate, anger). However, these are functional reactions to a documented physical assault, not cognitive distortions. The system correctly applied the exclusion criteria for Emotional Reasoning, distinguishing between feeling an emotion.

Case 4: Values/Preferences vs. Distortion

Text (Russian): Я не хочу детей. Не хочу тратить деньги на еду, одежду, игрушки. Не хочу нести ответственность. Я просто хочу попробовать жить для себя. Не хочу, чтобы они подверглись буллингу, как я в школе, потому что я знаю, что не смогу их правильно воспитать.

English Translation: I do not want children. I do not want to spend money on food, clothes, toys. I do not want to bear responsibility. I just want to try living for myself. I dont want them to be bullied like I was in school, because I know I wont be able to raise them properly.

- AI Classification: None
- Expert Annotation: None

The user expresses personal values and resource allocation preferences. While the tone is firm, there are no logical errors or distortions of reality. The model correctly distinguished a non-normative opinion from a cognitive distortion.

Case 5: Self-Referential Narrative vs. Personalization

Text (Russian): Я сегодня проснулся, приготовил себе завтрак и долго думал о том, что вы говорили в прошлом видео. Я начал регулярно практиковать предложенные упражнения по медитации и заметил, что мне действительно становится спокойнее в стрессовых ситуациях. Я очень благодарен за этот контент, потому что я давно искал что-то подобное для самопомощи. Я планирую продолжать заниматься и дальше, чтобы лучше понимать свои реакции.

English Translation: I woke up today, made myself breakfast and thought for a long time about what you said in the previous video. I started regularly practicing the suggested meditation exercises and noticed that I am indeed becoming calmer in stressful situations. I am very grateful for this content because I have been looking for something like this for self-help for a long time. I plan to continue practicing further to better understand my reactions.

- AI Classification: None
- Expert Annotation: None

Rationale: Although the text is entirely self-referential and uses the first-person pronoun (“I”) seven times, it does not contain Personalization. The author describes factual events (breakfast, watching a video), internal states (feeling calmer), and expressions of gratitude. There is no attribution of external events to one’s own character or irrational guilt. This case confirms that the classification protocol successfully distinguishes between a first-person narrative and the cognitive distortion of Personalization, ensuring that the hub status of Personalization in the network is not a linguistic artifact of self-referential discourse.

2.3 False Positives (Type I Error)

Case 6: Rhetorical Hyperbole vs. Catastrophizing

Text (Russian): Я и так очень плохо сплю, постоянно не высыпаюсь, в место того, что бы покушать на обеденном перерыве, я сплю. А если ребенок будет, я сдохну от нехватки сна. Чувствую, меня скоро до того доведут, что пойду рожу и оставлю им у порога.

English Translation: I already sleep very poorly, constantly sleep-deprived, instead of eating at lunch break, I sleep. And if I have a child, I will die from lack of sleep. I feel like they will soon drive me to the point where I go give birth and leave it at their doorstep.

- AI Classification: Catastrophizing
- Expert Annotation: None (Hyperbole)

The model triggered on the semantic marker die as a prediction of a catastrophic outcome. The expert identifies this as a common rhetorical hyperbole expressing exhaustion, not a literal belief in fatality. This illustrates the models tendency to interpret somatic metaphors literally.

Case 7: Idiom vs. Catastrophizing

Text (Russian): Так вот, она мне отвечает у меня подруга умерла. Я естественно поржала, потом написала Надеюсь ты шутишь. В итоге она не шутила и даже скинула статью, где писали про смерть подруги. У меня весь мир из-под ног ушел.

English Translation: So, she answers me my friend died. I naturally laughed, then wrote Hope you are joking. As it turned out, she wasn't joking and even sent an article about her friend's death. The whole world went out from under my feet.

- AI Classification: Catastrophizing
- Expert Annotation: None (Metaphor)

The phrase world went out from under my feet is a standard idiom for shock. The model interpreted it as a description of a global collapse or catastrophic event. Such false positives are mitigated in the final analysis by the high stability threshold required for network inclusion.

2.4 False Negatives (Type II Error)

Case 8: Implicit Mind Reading

Text (Russian): Эти люди в 56 лет без детей, никогда не признаются, что они жалеют, причем больше из принципа не признают. Это уже просто мода пошла не хотеть детей... так что ответы чаще не правдивые, а скорее всего больше для успокоения себя.

English Translation: These people aged 56 without children will never admit that they regret it, mostly out of principle. It is just a fashion now not to want children... so their answers are mostly untrue, likely just to comfort themselves.

- AI Classification: None
- Expert Annotation: Mind Reading

The user asserts certain knowledge of the internal emotional state (they regret) and hidden motivations (to comfort themselves) of a large group of strangers, directly contradicting those strangers explicit statements. The protocol defines this as Mind Reading (conviction in knowing thoughts without evidence). The AI missed this because it was phrased as a sociological observation rather than a direct telepathic claim (I know they think...).

Case 9: Implicit Fortune Telling

Text (Russian): Сначала 9 месяцев, роды, подорванное здоровье и не дай Бог осознание того что он мне не нужен... потом еще минимум 18 лет ответственности. Действительно похоже на тюремный срок. У меня еще вся жизнь впереди и тратить ее на то что написала выше! Увольте, ни за что!

English Translation: First 9 months, childbirth, ruined health, and God forbid realizing I don't need him... then at least 18 more years of responsibility. It really looks like a prison term. I have my whole life ahead of me and to spend it on what I wrote above! Spare me, no way!

- AI Classification: None
- Expert Annotation: Fortune Telling, Catastrophizing

The text predicts a specific negative scenario (ruined health, prison term) as an inevitable outcome of a hypothetical event. Under the protocol, this qualifies as Fortune Telling (certainty in negative

outcome) and Catastrophizing. The AI likely interpreted this as a rhetorical expression of personal preference/values (Childfree stance) rather than a cognitive distortion of probability, adhering to the literal interpretation constraint too strictly.

3 SUPPLEMENTARY TABLES AND FIGURES

3.1 Figures

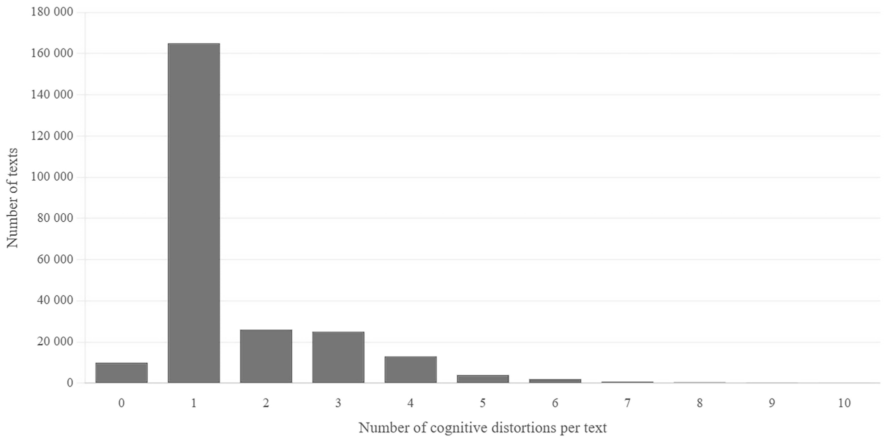

Figure S1: Distribution of cognitive distortions per text in Russian-language corpus

3.2 Tables

Table S1. Frequency Distribution of Cognitive Distortions (Total Instances = 443,447)

Rank	Cognitive Distortion	Frequency	Percentage
1	All-or-Nothing Thinking	68592	15.5%
2	Overgeneralization	62843	14.2%
3	Catastrophizing	50505	11.4%
4	Discounting Positive	44113	10.0%
5	Labeling	34031	7.7%
6	Personalization	32474	7.3%
7	Mental Filter	28776	6.5%
8	External Locus of Control	25766	5.7%
9	Emotional Reasoning	23601	5.3%
10	Tunnel Vision	15863	3.6%
11	Mind Reading	13554	3.1%
12	Should Statements	13104	3.0%
13	Social Comparison	10150	2.3%
14	Fortune Telling	7579	1.7%
15	Rationalization	4992	1.1%
16	Rumination	3165	.7%
17	Learned Helplessness	2568	.6%
18	Fairness Fallacy	1771	.4%

	1	2	3	4	5	6	7	8	9	10	11	12	13	14	15	16	17	18
1. Catastrophizing	50 505	27 020	20 377	16 618	11 880	9 834	10 019	3 569	3 473	2 012	1 178	3 823	495	2 050	646	941	171	115
2. All-or-Nothing Thinking	27 020	68 592	28 134	22 530	14 012	11 279	16 287	4 666	4 302	3 163	1 965	4 662	915	2 803	984	1 684	238	137
3. Overgeneralization	20 377	28 134	66 876	15 587	10 012	12 618	13 830	3 038	3 591	2 512	1 721	4 390	792	1 511	698	1 261	273	143
4. Discounting the Positive	16 618	22 530	15 587	44 113	8 653	7 747	5 548	2 444	2 095	1 335	909	3 161	436	1 141	642	1 088	124	88
5. Personalization	11 880	14 012	10 012	8 653	32 474	6 131	4 667	1 579	1 456	1 960	547	2 249	333	1 763	512	635	109	79
6. Mental Filter	9 834	11 279	12 618	7 747	6 131	29 502	3 650	647	1 224	1 014	848	1 364	244	539	381	476	141	56
7. Labeling	10 019	16 287	13 830	5 548	4 667	3 650	34 031	1 988	2 287	1 217	834	2 601	433	1 909	329	749	110	57
8. External Locus of Control	3 569	4 666	3 038	2 444	1 579	647	1 988	25 766	448	268	269	437	175	552	149	248	57	47
9. Emotional Reasoning	3 473	4 302	3 591	2 095	1 456	1 224	2 287	448	23 601	481	272	519	146	434	129	181	36	34
10. Should Statements	2 012	3 163	2 512	1 335	1 960	1 014	1 217	268	481	13 104	228	643	138	546	126	180	41	31
11. Fortune Telling	1 178	1 965	1 721	909	547	848	834	269	272	228	7 579	374	116	338	98	158	37	29
12. Tunnel Vision	3 823	4 662	4 390	3 161	2 249	1 364	2 601	437	519	643	374	15 863	160	426	151	217	40	51
13. Rationalization	495	915	792	436	333	244	433	175	146	138	116	160	4 992	149	35	84	28	28
14. Mind Reading	2 050	2 803	1 511	1 141	1 763	539	1 909	552	434	546	338	426	149	13 554	174	174	43	28
15. Rumination	646	984	698	642	512	381	329	149	129	126	98	151	35	174	3 165	56	30	32
16. Social Comparison	941	1 684	1 261	1 088	635	476	749	248	181	180	158	217	84	174	56	10 150	47	33
17. Fallacy of Fairness	171	238	273	124	109	141	110	57	36	41	37	40	28	43	30	47	1 771	18
18. Learned Helplessness	115	137	143	88	79	56	57	47	34	31	29	51	28	28	32	33	18	2 568

0 — 70,000 **Co-occurrence Frequency**

Figure S2: Complete cognitive distortion network (N=18) showing all analyzed distortions

Table S2. Sensitivity Analysis: Network Stability Under Simulated Annotation Errors (N=249,414 texts, k=50 iterations per noise level)

Noise Level	Nodes M (SD)	Edges M (SD)	Density M (SD)	Clustering M (SD)	Hub Jaccard M (SD)	Interpretation
0% (baseline)	13.0 ± 0.0	35.0 ± 0.0	0.449 (.000)	0.598 (.000)	1.000 (.000)	Ground truth
5%	13.2 ± 0.5	34.2 ± 0.5	0.424 (.028)	0.515 (.026)	1.000 (.011)	Stable
10%	13.0 ± 0.0	34.0 ± 0.0	0.436 (.000)	0.525 (.000)	1.000 (.000)	Stable
15%	15.0 ± 0.0	69.8 ± 1.4	0.665 (.014)	0.849 (.023)	0.983 (.073)	Stable
20%	15.8 ± 0.4	70.6 ± 1.1	0.604 (.029)	0.827 (.031)	0.979 (.080)	Stable
25%	16.0 ± 0.2	72.4 ± 1.2	0.607 (.018)	0.831 (.029)	0.968 (.098)	Stable
30%	16.0 ± 0.0	75.2 ± 1.2	0.627 (.010)	0.866 (.006)	0.693 (.099)	Degrading
35%	16.0 ± 0.0	75.2 ± 1.2	0.627 (.010)	0.867 (.006)	0.694 (.103)	Degrading
40%	16.0 ± 0.0	87.8 ± 1.8	0.732 (.015)	0.889 (.010)	0.454 (.124)	Degraded
45%	16.0 ± 0.0	91.2 ± 1.8	0.760 (.015)	0.897 (.009)	0.378 (.093)	Degraded
50%	16.0 ± 0.0	95.2 ± 1.4	0.793 (.012)	0.891 (.004)	0.340 (.095)	Collapse

Note. Hub Jaccard = overlap with original top-5 hubs. Noise simulates both false positives (incorrect label additions) and false negatives (missed labels). Stability threshold: Hub Jaccard $\geq$ 0.60 indicates preservation of core structure. Original network: 13 nodes, 35 edges, density=0.449, clustering=0.525. Network remains stable up to 25% noise (Hub Jaccard $\geq$ 0.97), begins degrading at 30% (Hub Jaccard=0.693), and collapses beyond 40% (Hub Jaccard $<$ 0.50). Density increase under noise reflects false positive connections rather than core-periphery contraction.

Table S3. Prevalence and Bootstrap Stability of Cognitive Distortions. Distortions are ranked by frequency.

Cognitive Distortion	Prevalence (%)	Stability (%)
All-or-Nothing Thinking	27.5	100.0
Overgeneralization	25.2	100.0
Catastrophizing	20.2	100.0
Discounting Positive	17.7	100.0
Labeling	13.6	100.0
Personalization	13.0	100.0
Mental Filter	11.4	100.0
External Locus of Control	10.3	0.0
Emotional Reasoning	9.5	0.0
Tunnel Vision	6.4	100.0
Mind Reading	5.4	98.6
Should Statements	5.3	100.0
Social Comparison	4.1	0.0
Fortune Telling	3.0	60.7
Rationalization	2.0	9.5
Rumination	1.3	100.0
Learned Helplessness	1.0	60.2
Fairness Fallacy	0.7	92.7

Note: Prevalence indicates the percentage of texts containing the distortion. Stability reflects the frequency of the node's inclusion in the network (Lift $>$ 1.0) across 10,000 bootstrap iterations. Learned Helplessness (60.2%) was excluded despite moderate stability because it did not demonstrate significant lift ($>$1.0) in the full observed dataset.

Table S4. Cross-source validation of network stability across 10,655 distinct video sources. The dataset was partitioned into "High-Activity" and "Low-Activity" groups using three data-driven thresholds to test structural invariance.

	High-Activity Group		Low-Activity Group		
Method	Sources (N)	Texts (N)	Sources (N)	Texts (N)	Correlation (r)
Median Split	6,077	39,208	4,578	4,578	0.751
Pareto (Top 20%)	2,131	27,768	8,524	16,018	0.801
Top 25% Split	2,663	30,281	7,992	13,505	0.803
